# Excess Mortality in Patients with Severe Mental Disorders in 1996-2010 in Finland

**DOI:** 10.1371/journal.pone.0152223

**Published:** 2016-03-24

**Authors:** Sonja Lumme, Sami Pirkola, Kristiina Manderbacka, Ilmo Keskimäki

**Affiliations:** 1 Department of Health and Social Care Systems, Health and Social Systems Research Unit, National Institute for Health and Welfare, Helsinki, Finland; 2 School of Health Sciences, University of Tampere, Tampere, Finland; Hunter College, UNITED STATES

## Abstract

Unselected population-based nationwide studies on the excess mortality of individuals with severe mental disorders are scarce with regard to several important causes of death. Using comprehensive register data, we set out to examine excess mortality and its trends among patients with severe mental disorders compared to the total population. Patients aged 25–74 and hospitalised with severe mental disorders in 1990–2010 in Finland were identified using the national hospital discharge register and linked individually to population register data on mortality and demographics. We studied mortality in the period 1996–2010 among patients with psychotic disorders, psychoactive substance use disorders, and mood disorders by several causes of death. In addition to all-cause mortality, we examined mortality amenable to health care interventions, ischaemic heart disease mortality, disease mortality, and alcohol-related mortality. Patients with severe mental disorders had a clearly higher mortality rate than the total population throughout the study period regardless of cause of death, with the exception of alcohol-related mortality among male patients with psychotic disorders without comorbidity with substance use disorders. The all-cause mortality rate ratio of patients with psychotic disorders compared to the total population was 3.48 (95% confidence interval 2.98–4.06) among men and 3.75 (95% CI 3.08–4.55) among women in the period 2008–10. The corresponding rate ratio of patients with psychoactive substance use disorders was 5.33 (95% CI 4.87–5.82) among men and 7.54 (95% CI 6.30–9.03) among women. Overall, the mortality of the total population and patients with severe mental disorders decreased between 1996 and 2010. However, the mortality rate ratio of patients with psychotic disorders and patients with psychoactive substance use disorders compared to the total population increased in general during the study period. Exceptions were alcohol-related mortality among patients with psychoactive substance use disorders and female patients with psychotic disorders, as well as amenable mortality among male patients with psychotic disorders. The mortality rate ratio of persons with mood disorders compared to the total population decreased. The markedly high mortality amenable to health care intervention among patients with severe mental disorders found in our study suggests indirectly that they may receive poorer quality somatic care. The results highlight the challenges in co-ordinating mental and somatic health services.

## Introduction

Research has repeatedly shown disparities in health and health care between those with mental disorders and the general population. Individuals with severe mental disorders (SMD) have greater mortality and increased risk of premature death [1, 2]. Individuals suffering from any of the common mental disorders, especially persons with psychotic disorders, major depressive disorders, and substance use disorders, have increased risk of premature death. The risk is not similar for all mental health problems and the underlying causes of death may also vary [3, 4]. Patients with psychotic disorders are also highly comorbid with alcohol and other substance abuse disorders [5, 6]. Psychiatric patients comorbid with substance use disorders have a higher risk of mortality than psychiatric patients without substance use disorders according to earlier studies [3, 7].

Higher risk of suicides, unintentional injuries, less healthy lifestyles, poorer socioeconomic circumstances, adverse metabolic effects of antipsychotic medications, and social consequences of mental illness have been found to partly explain these findings [3, 8, 9]. The excess mortality is also a consequence of increased risk of comorbid somatic illnesses and higher case-fatality among persons with SMDs [3, 10].

Despite reforms in mental health services, the mortality gap has remained in many Western countries, which generally have well-functioning health systems [11–13]. An important factor contributing to this gap is also poorer access to and quality of somatic care [1, 3, 14, 15]. Studying amenable deaths provides information that can be used to evaluate health care performance. Amenable mortality captures premature deaths that would have been prevented by timely and effective health care intervention and is increasingly used as an indicator of the effectiveness of health care [16]. Mortality amenable to medical intervention consists of selected causes of deaths, focusing on conditions for which effective clinical interventions exist generally in people aged less than 75 years. Age limits vary for some diseases due to the fact that health care may not be able to contribute substantially to survival above or below a certain age. This definition of amenable mortality was first proposed 40 years ago by Rutstein et al. (1976) and has been adopted and modified in several studies [16–18].

Several studies have examined excess amenable mortality among psychiatric patients in different study settings, suggesting that somatic care provided to patients with mental illnesses is less adequate than for the population in general [3, 19–21]. Although earlier studies address excess mortality in patients with SMDs, representative/unselected population-based nationwide studies are scarce in regard to several important causes of death, such as alcohol-related deaths and amenable deaths. In addition, there is a gap in the literature when it comes to focusing on time trends in excess mortality.

Thus, using comprehensive register data, we aim to examine excess mortality and its trends among patients with SMDs in Finland in 1996–2010 compared to the total population. Mortality among patients hospitalised due to psychotic disorders, psychoactive substance use disorders and mood disorders is studied by several causes of death groups. Our aim is also to assess the quality of somatic care provided to patients with SMDs by using mortality amenable to health care as an indicator.

## Material and Methods

### Data

The cohort of patients with severe mental disorders aged 25–74 was defined as those hospitalised at least once with the main diagnosis being a psychotic disorder (ICD-10: F20-F29), a psychoactive substance use disorder (F10-F19), or a mood disorder (F30-F39) during the period 1990–2010 in Finland and alive on January 1^st^ 1996. The hospitalisations were identified using the national hospital discharge register, which includes all hospital admissions in Finland. The data on hospital care were individually linked to the population registers of Statistics Finland for mortality data and demographic factors for the years 1996–2010. The study protocol was approved by the Ethical Review Board of the National Institute for Health and Welfare. Permission to use the above-described register data was obtained from the competent authorities. The data compilation was conducted by the competent authorities, with the study group receiving anonymised data.

### SMD categories

The study population consists of all non-institutionalised patients with SMDs and resident in Finland. Persons aged over 25 years remain in the study population while aged under 75 years and until the end of the study period, death or moving to another SMD category. We divided the cohort of patients with SMDs into three diagnosis categories: 1) psychotic disorders (PD), 2) psychoactive substance use disorders (PSD), and 3) mood disorders (MD) and analysed the data in these categories due to the different nature of the mental disorders in question. Using the same hierarchical rule as in an earlier Finnish study [22], we categorised the individual as a patient with a psychotic disorder if he/she had been hospitalised with a PD in addition to either of the two other disorders. Following this rule further, an individual with a hospitalisation due to both a PSD and a MD was categorised as a patient with PSD. Thus, the patients with MDs in our study did not have any record of PDs or PSDs. Patients were defined as belonging to the category from the first admission date for the diagnosis at issue. Patients with PDs and who were comorbid with any of the other SMDs in question belonged into the PSD or MD category until the first admission due to PD and remained in the PD category until the end of the follow-up. The total non-institutionalised Finnish population aged 25–74 in 1996–2010 was used as the comparison group. The data were analysed as tabulated, i.e. the number of deaths by person years in the follow-up of the corresponding population (SMD category or total population) by five-year age groups and gender.

### Mortality groups

We studied excess mortality among patients with SMDs compared to the total population in 1996–2010. In addition to all-cause mortality, we classified mortality into sub-groups: mortality amenable to health care interventions, ischaemic heart disease (IHD) mortality, disease mortality, and alcohol-related mortality (See Table 1 for a list of causes of death). In this study the selection of causes of death considered amenable to health care was modified from classifications used by Nolte and McKee (2008) and Page et al. (2006) [18, 23]. Those underlying causes of death caused directly by excess alcohol consumption or diseases caused by excess alcohol consumption where defined as alcohol-related deaths. The sub-groups are not, however, totally exclusionary. For example, IHD deaths are included in the disease mortality group.

**Table 1:** List of causes of death.

Cause of death group	ICD-10	
***Disease mortality***	A00- R99, excl. R999	
***Ischaemic heart disease mortality***	I20–25	
***Alcohol-related mortality***		
Mental and behavioural disorders due to psychoactive use substance use	F10	
Degeneration of nervous system due to alcohol	G31.2	
Epileptic seizures related to alcohol	G40.51	
Alcoholic polyneuropathy	G62.1	
Alcoholic myopathy	G72.1	
Alcoholic cardiomyopathy	I42.6	
Alcoholic gastritis	K29.2	
Alcoholic liver disease	K70	
Alcohol induced acute pancreatitis	K85.2, K86.0	
Maternal care for (suspected) damage to fetus from alcohol	O35.4	
Fetus and newborn affected by maternal use of alcohol	P0.43	
Fetal alcohol syndrome (dysmorphic)	Q86.0	
Accidental poisoning by and exposure to alcohol	X45	
***Mortality amenable to health care interventions***		**Age**^a^
Diphtheria, Tetanus, Poliomyelitis, and Varicella	A35–36, A80, B01	1–74
Rubella	B06	1–74
Scarlatina	A38	1–74
Meningococcus	A39	1–74
Erysipelas	A46	1–74
Legionellosis	A48.1	1–74
Malaria	B50–54	1–74
Streptococcal pharyngitis	J02.0	1–74
Cellulitis	L03	1–74
Tuberculosis	A15–19, B90	1–74
Malignant neoplasm of colon and rectum	C18–21	1–74
Melanoma of skin	C43	1–74
Malignant neoplasm of skin	C44	1–74
Malignant neoplasm of breast	C50	1–74
Malignant neoplasm of cervix uteri	C53	1–74
Malignant neoplasm of cervix uteri and body of uterus	C54–55	1–44
Malignant neoplasm of bladder	C67	1–74
Benign tumors	D10–36	1–74
Hypertensive disease	I10–13, I15	1–74
Cerebrovascular disease	I60–69	1–74
Diseases of the thyroid	E00–07	1–74
Diabetes mellitus	E10–14	1–49
Epilepsy	G40–41	1–74
Asthma	J45–46	15–49
COPD	J40–44	15–49
Septicaemia	A40–41	1–74
Malignant neoplasm of testis	C62	1–74
Hodgkin’s disease	C81	1–74
Leukaemia	C91–95	1–44
Rheumatic and other valvular heart disease	I01–09	1–74
Influenza	J09–11	1–74
Pneumonia	J12–18	1–74
Peptic ulcer	K25–28	1–74
Appendicitis	K35–38	1–74
Abdominal hernia	K40–46	1–74
Cholelithiasis and cholecystitis	K80–81	1–74
Nephritis, nephrosis, and nephropathy	N00–09, N17–19, N25–27	1–74
Obstructive uropathy and prostatic hyperplasia	N13, N20–21, N35, N40	1–74
Maternal death	O00–O99	All
Congenital cardiovascular anomalies	Q20–Q28	1–74

^a^ Age restrictions are only for mortality amenable to health care interventions. In our study, deaths of people aged under 25 years were excluded from the analyses.

### Statistical methods

To study the overall level of mortality in Finland, we calculated annual age-standardised mortality rates per 100 000 person years among the total population in 1996–2010 by cause-of-death groups. We used the direct method of standardisation and as the standard population we used the non-institutionalised Finnish population in 2010. To ensure a sufficient number of events in each age-stratum and to avoid random variation, we combined the annual data into five three-year periods (1996–98, 1999–2001, 2002–04, 2005–07, and 2008–10) when studying the excess mortality of the patients with SMDs by categories. The excess mortality was then estimated using the rate ratio (RR) approach by comparing mortalities in the three SMD categories with mortality in the total population. The 95% confidence intervals for the rate ratio of two standardised rates were estimated using Gaussian approximation [24].

We performed additional analyses for persons with PDs and excluded patients comorbid with PSD admissions, since persons with PDs are highly comorbid with alcohol and other substance abuse disorders, which of itself also increases the risk of premature death.

Furthermore, we conducted sensitivity analyses for the mortality rate ratios for patients with SMDs versus the total population by including only patients with hospitalisations due to SMDs from a preceding period of five years or during the particular study year. This enabled us to study whether the longer follow-up times for the later study periods had an influence on the results.

SAS (SAS Institute Inc., Cary, NC, USA) version 9.3 was used in these analyses.

## Results

### Background characteristics

The average annual number of person years in the study period for the non-institutionalised total population aged 25–74 was about 1 598 000 among men and 1 637 000 among women. The proportion of the patient cohort hospitalised due to severe mental health disorders of the total population was 4% among men and 3% among women in 1996 and was somewhat higher in 2010. The majority (over 50%) of male patients with SMDs were categorised as patients with PSDs, and the majority of female patients with SMDs were categorised as patients with PDs according to our definitions (Table 2). During the study period, the proportions of male patients with PDs and PSDs had decreased somewhat and the proportion of MDs had increased from 14% to 23%. Among women, the proportion of patients with PDs had clearly decreased (from 49% to 34%), while the proportion of MDs had increased from 30% to 43%. In 1996, 63% of all male patients with SMDs had hospitalisations due to PSDs and in 2010 the proportion was 57%, whereas among female patients this proportion was much less, at 26% both in 1996 and 2010. Among male patients with PDs, 23% also had hospitalisations due to PSDs in 1996 and slightly less in 2010. Among female patients with psychotic disorders, the proportion of hospitalisations due to PSDs was much lower than among male patients, at less than 10% in both years.

**Table 2:** Number of person years (py) for the total population and for patients with severe mental disorders (SMD), non-institutionalised and aged 25–74, the proportion of patients with SMDs by hierarchical categories (patients with psychotic disorders [PD], psychoactive substance use disorders [PSD] or mood disorders [MD]), and the proportion of patients with hospitalisations due to PSDs by gender in 1996 and 2010 in Finland.

	**MEN**											
	**year 1996**						**year 2010**					
	**Total population**	** **	** **	**SMD**	→	63% PSD	**Total population**	** **	** **	**SMD**	→	57% PSD
py	1,564,800			57,500	** **		1,650,100			91,400		** **
			↙↓↘					↙↓↘				
		**PD**	** **	**PSD**	** **	**MD**		**PD**	** **	**PSD**	** **	**MD**
		30%		56%		14%		25%		52%		23%
		↓						↓				
		23% also PSD						19% also PSD				
												
	**WOMEN**											
	**year 1996**						**year 2010**					
	**Total population**	** **	** **	**SMD**	→	26% PSD	**Total population**	** **	** **	**SMD**	→	26% PSD
py	1,623,300			40,600		** **	1,677,900			71,800		** **
		↙↓↘						↙↓↘				
		**PD**	** **	**PSD**	** **	**MD**		**PD**	** **	**PSD**	** **	**MD**
		49%		21%		30%		34%		23%		43%
		↓						↓				
		9% also PSD						9% also PSD				

↙↓↘ Categorised according to the hierarchial rules described in the Manuscript.

→ Proportion of patients hospitalised with PSDs among patients with SMDs.

↓ Proportion of patients hospitalised with PSDs among patients with PDs.

### Mortality rates

**Fig 1:**
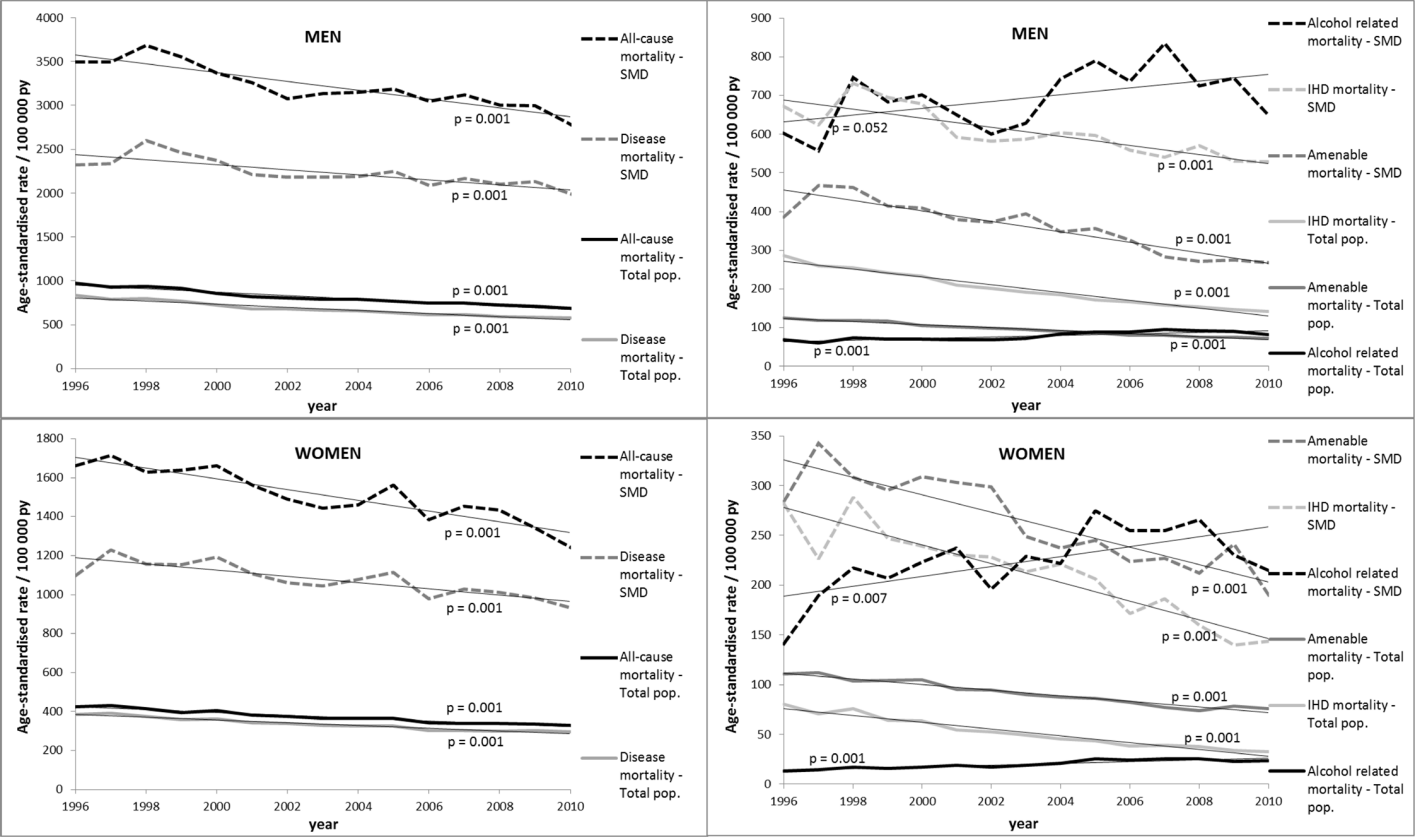
Age-standardised mortality rates (/100 000 person years) among the total population and patients with severe mental disorders (SMD) by gender and cause-of-death groups for the period 1996–2010 in Finland. The data for Fig 1 are available in S1 Dataset.

Fig 1 presents age-standardised mortality rates (/100 000 person years) by cause-of-death groups *among the total population* and *patients with SMDs* aged 25–74 by cause-of-death groups for the period 1996–2010 in Finland. In 1996, the all-cause mortality rate among the total population was 980 among men and 420 among women and decreased by 30% (men) and 20% (women) during the study period (p-values for linear trend = 0.001). The mortality trends for all causes-of-death sub-groups were downward (p = 0.001) except for alcohol-related mortality, which increased from 68 to 81 among men and from 13 to 23 among women (p = 0.001). Similarly, mortality among patients with SMDs decreased regardless of cause of death (p = 0.001), except for alcohol-related mortality, which increased (p = 0.052 for men, p = 0.007 for women). The all-cause mortality rate among patients with SMDs was 3500 among men and 1700 among women in 1996 and decreased by 20% (men) and 25% (women) in the study period. When studying the SMD categories separately, differences were found in alcohol-related mortality, which increased significantly only among male patients with PDs (p = 0.001). Among female patients with PDs and all patients with PSDs the increasing trend was not statistically significant (p > 0.05). Among male patients with MDs, alcohol-related mortality decreased significantly (p = 0.014), but among female patients with MDs the decreasing trend was not significant (p = 0.837).

### Excess mortality

**Fig 2:**
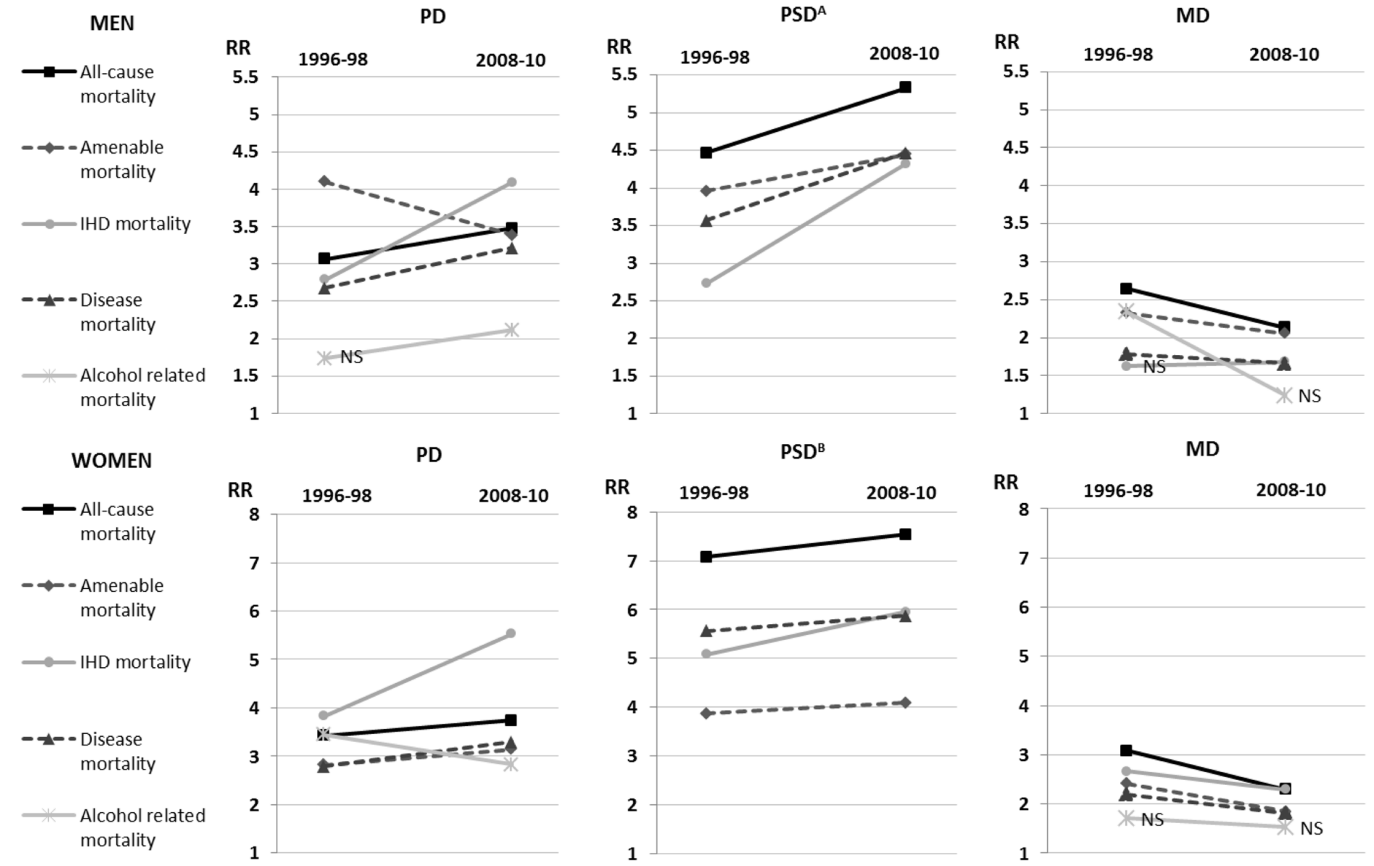
The rate ratios (RR) of the excess mortality in patients with severe mental disorders (SMD) compared to the total population by gender, cause-of-death groups, and the SMD categories (patients with psychotic disorders [PD], psychoactive substance use disorders [PSD], and mood disorders [MD]) in the periods 1996–98 and 2008–10 in Finland. (A) The alcohol-related mortality RR was 15.18 (95% confidence interval 12.16–18.96) in the period 1996–98 and 13.30 (95% CI 11.28–15.69) in the period 2008–10. (B) The alcohol-related mortality RR was 54.79 (95% CI 34.22–87.71) in the period 1996–98 and 35.78 (95% CI 25.76–49.71) in the period 2008–10. Non-significant RRs indicated as NS. The data for Fig 2 in addition to the data for the other study periods and all 95% confidence intervals are available in S2 Dataset.

Next we studied the excess mortality in patients with SMDs compared to the total population by disease categories in five three-year periods (Fig 2). The change in the excess mortality was mainly linear, thus we present results only for the first and the last study periods. Generally, women had higher excess mortality estimated with the rate ratios of age-standardised mortality rates (RR).

### Patients with psychotic disorders

The excess mortality among *patients with PDs* was statistically significant in all causes-of-death groups, except that the RR for alcohol-related mortality was not significant in the period 1996 to 2004 among men. The excess mortality increased during the study period with the exception of amenable mortality among men and alcohol-related mortality among women. In the period 2008–10, the RR for all-cause mortality was 3.48 (95% confidence interval 2.98–4.06) among men and 3.75 (95% CI 3.08–4.55) among women (Fig 2).

### Patients with psychoactive substance use disorders

In general, *patients with PSDs* had the highest excess mortality of the three SMD categories (Fig 2). Even though the alcohol-related excess mortality decreased during the study period, it was especially high throughout the study period and the RR was as high as 13.30 (95% CI 11.30–15.70) among men and 35.80 (95% CI 25.80–49.70) among women in 2008–10. The excess mortality increased in all the other causes of death groups during the study period. In 2008–10, the RR for all-cause mortality was 5.33 (95% CI 4.87–5.82) among men and 7.54 (95% CI 6.30–9.03) among women.

### Patients with mood disorders

*Patients with MDs* had the lowest excess mortality among both genders (Fig 2). In general, the excess mortality in patients with MDs decreased in the period 1996 to 2010. Especially, alcohol-related mortality decreased from 2.34 (95% CI 1.09–5.02) to 1.24 (95% CI 0.62–2.46) among men. In 2008–10, the RR for all-cause mortality was 2.14 (95% CI 1.75–2.61) among men and 2.30 (95% CI 1.84–2.88) among women.

### Sensitivity analyses

The sensitivity analyses yielded systematically higher rate ratios for the mortality differences between patients with SMDs and the total population compared to the original RRs in each three-year-period. In these analyses we included only patients with hospitalisations due to SMDs from a preceding period of five years or during the particular study year. Only RRs for alcohol-related mortality in three study periods among women were found to be somewhat lower compared to the original RRs. The data for the sensitivity analyses are available in S3 Dataset.

### Impact of hospitalisations due to PSDs among patients with psychotic disorders

**Fig 3:**
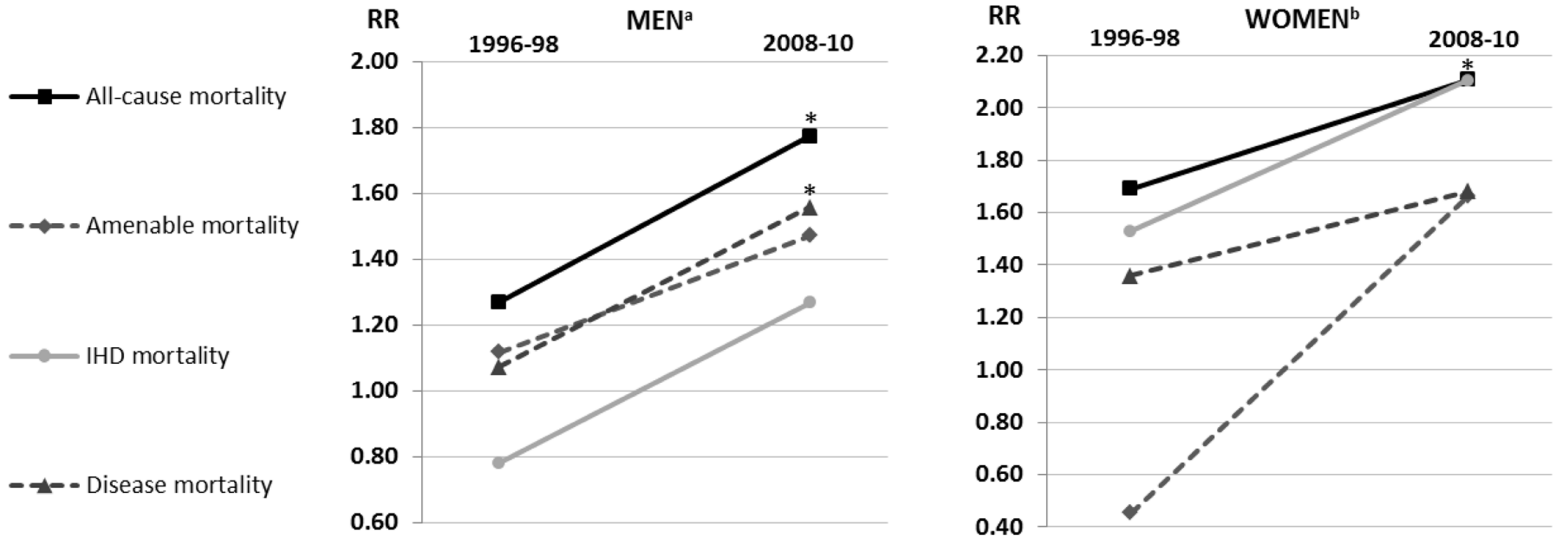
The rate ratios (RR) of the excess mortality in patients with psychotic disorders (PD) comorbid with psychoactive substance use disorders (PSD) compared to patients with PDs without PSDs by gender and cause-of-death groups in the periods 1996–98 and 2008–10 in Finland. (a) The alcohol-related mortality RR was 5.70 (95% confidence interval 1.46–22.26) in the period 1996–98 and 11.44 (95% CI 3.81–34.22) in the period 2008–10. (b) The alcohol-related mortality RR was 22.87 (95% CI 3.18–164.66) in the period 1996–98 and 11.36 (95% CI 2.64–48.84) in the period 2008–10. Significant RRs indicated as *. The data for Fig 3 in addition to the data for the other study periods and all 95% confidence intervals are available in S4 Dataset.

We also studied the impact of hospitalisations due to PSDs on the excess mortality among patients with PDs (Fig 3). We found that among patients with co-existing PSD, only alcohol-related mortality was significantly higher compared to patients without PSDs among patients with PDs in 1996–98. The alcohol-related mortality rate was 5.70-fold higher (95% CI 1.46–22.26) among men and 22.87-fold higher (95% CI 3.18–164.66) among women with co-existing PSD in 1996–98. On the other hand, IHD mortality was higher among male patients without PSDs and amenable mortality was higher among female patients without PSDs in 1996–98. During the study period the differences increased somewhat and in 2008–10 also disease and all-cause mortality were significantly higher for those with comorbidity with PSD among men. Among women, all-cause mortality became significantly higher by 2008–10 for those with co-existing PSDs. Among them, differences in alcohol-related mortality decreased, but the RR remained still notably high, at 11.36 (95% CI 3.18–164.66). Throughout the study period alcohol-related mortality was even lower among male patients with PDs without comorbidity with PSD compared to the total male population, but the differences were not statistically significant.

## Discussion

Our study showed that compared to the overall population in Finland the patients with severe mental disorders have clearly higher mortality regardless of cause of death, with the exception of male patients with psychotic disorders without comorbidity with psychoactive substance use disorders. We studied the excess mortality by three common SMD categories: psychotic disorders, psychoactive substance use disorders, and mood disorders. In general, patients with PSDs had the highest excess mortality according to our findings, which is in line with earlier studies [3]. In addition to the excess all-cause mortality, we examined the excess mortality in several of the causes-of-death sub-groups in which previous studies have found high mortality among patients with SMDs. Earlier studies, however, have not examined in detail the excess alcohol-related mortality (not only cirrhosis) of patients with SMDs. Our study also adds to the literature by exploring trends in the excess mortality in Finland using a population-based dataset covering a long time period. During the study period 1996–2010 there was a clear decrease in all-cause mortality in the total population and in patients with SMDs. However, we found that the relative differences between patients with the most severe forms of mental disorders, PDs and PSDs, increased compared to the total population, while the excess mortality of persons with MDs somewhat decreased.

In the current study, the excess mortality in patients hospitalised with SMD was found to be systematically pronounced throughout the long study period and for each cause-of-death group. As noted in other studies, this finding is likely to partly reflect differences in health behaviours. Patients with mental disorders have higher smoking rates, poorer diet, are less physical active, and suffer more from obesity than the general population [25]. One notable risk factor concerning health behaviours is heavier use of alcohol and drugs, which was also found in our study; about 60% of the studied male patients and a quarter of the female patients with SMDs had hospitalisations due to substance abuse. The most serious consequence of substance abuse was clearly seen from our results: among patients with PSDs the excess mortality was notably high in each cause-of-death group. Even though substance abuse was much more common among men than among women, alcohol-related mortality as a consequence of abuse was substantially high among female patients with PDs or PSDs.

Psychiatric patients comorbid with a substance use disorder have a higher risk of mortality than psychiatric patients without a substance use disorder according to earlier studies [3, 7]. Thus, we also explored the impact of comorbidity with PSD on the excess mortality of patients with psychotic disorders. We found that comorbidity with PSD had a strong influence only on alcohol-related mortality among patients with PDs. In some study periods it also had a significant, but rather weak effect on disease mortality. Interestingly, male patients with PDs without comorbidity with PSDs had even lower alcohol-related mortality than the total population throughout the study period (the differences were not, however, statistically significant). It is possible that among at least certain sub-populations without a comorbid PSD, alcohol use patterns are not particularly hazardous or risky. Among persons with severe mental illness, deficits in executive functioning may protect from impulsive alcohol use patterns. Differences in all-cause mortality in the later years of the study period were explained by differences in alcohol-related mortality among both genders.

The excess mortality of patients hospitalised with SMDs is also likely to partly reflect differences in poorer physical health; among patients with psychosis this often occurs at an early age. Many chronic diseases, such as diabetes, acute respiratory disorders, and asthma, are common among the mentally ill. In particular, several studies have shown a high prevalence of cardiovascular diseases among patients with psychosis [26, 27]. The explanations for elevated risk for morbidity are complex and interconnected, and relate to a range of external influences of both risk and (a lack of) protective factors. For example, social exclusion and lack of social networks, which are also risk factors for poorer physical health, are common among patients with SMDs. According to our findings, excess disease mortality was systematically and significantly high among both genders, which is obviously caused by higher chronic disease prevalence among patients with SMDs. Moreover, the excess IHD mortality was notably high among female patients with PDs or PSDs. Lawrence et al., Kisely et al., and Manderbacka et al., for example, suggest an explanation for the excess IHD mortality: Patients with SMDs may suffer from lower quality of and poorer access to treatment of cardiovascular diseases [14, 19, 22].

The excess mortality of patients with SMDs is undoubtedly also a consequence of differences in care seeking for somatic diseases. However, since this patient group is already in touch with health services (at least in settings where the patient group is identified from hospital registers) due to their mental health problems, it may also partly reflect the availability and quality of somatic health services. During the 1990s and early 2000s a large part of mental health services were administratively transferred to health centres and many long-term patients were transferred from hospital care to outpatient care or to transitional services, e.g. supported housing in Finland. Institutional care is provided in the psychiatric units of hospitals and while some of the units are physically located in general hospitals, some are separate psychiatric hospitals [28]. In these separate hospital settings, patients with SMDs may have some difficulties accessing adequate somatic care and their somatic symptoms may not be recognised.

Disparities in amenable mortality are used as a warning signal for the quality of care. Ringbäck Weitoft et al. were one of the first research groups to study excess amenable mortality among psychiatric patients compared to the general population [29]. They found markedly elevated risks for the period 1986–90. Several studies using different settings and classifications have since concordantly reported increased risk of amenable mortality for psychiatric patients [3, 20, 21]. The main results of the current study are in line with this finding. However, one notable result in our study was that the excess amenable mortality of female patients with PDs comorbid with PSDs compared to the total female population was not significant in 1996–98, but increased later and was about five times higher in all the other study periods.

Regarding the decreasing excess mortality among the MD category, a relative change in the characteristics of MD patients treated in hospital is possible given the overall shortened treatment periods and more numerous kinds of inpatients [30]. This may include more patients with lower risk for diseases and death. Another explanation could be improved management of depression, which is supported by a remarkable decrease in suicide mortality in Finland within the last decades.

We also conducted sensitivity analyses for the excess mortality rate ratios by including only patients with hospitalisations due to SMDs from a preceding period of five years or during the follow-up year. These sensitivity analyses yielded systematically higher rate ratios. In addition, the rate ratios of the sensitivity analyses were consistently higher throughout the study period compared to the original results. The results corroborated with our main findings. These sensitivity analyses enabled us to assure that the definition of the cohort and cumulating follow-up time did not affect the results. Severe mental disorders have often long natural courses, thus the results are not sensitive to the definition of the population at risk, as can be seen from our results.

Health register data in Finland are of high quality in general [31]. Thus, a major strength of our study is that we were able to use individual-level register data on hospital use among all Finnish residents aged 25 to 74 years from a 21-year period linked with the causes-of-death statistics. The accuracy and coverage of the Hospital Discharge Register has been reported to be generally good [32]. Also, Finnish data on mortality are comprehensive due to the completeness of death registration, the process for expert review of disputed cases, and the high autopsy rate for deaths from suspicious and external causes [33].

Amenable mortality is a crude indicator of access to care; thus the results should be interpreted with caution. While the strength of amenable mortality is that it relies on a list of conditions in which death could be avoided by identifiable effective interventions in health care, it simultaneously sums up death rates from a long list of diseases, some of which have a negligible contribution to amenable mortality. The mortality of patients hospitalised due to mental health disorders compared to mental health outpatients is higher. Consequently, our results may not be applicable to all individuals with mental health disorders. In addition, we were not able to study suicides separately.

## Conclusions

Studying mortality rates and the excess mortality of patients hospitalised with SMDs may reflect the availability of health services, since this patient group is already in touch with health services due to their mental health problems. Our results of notably high excess amenable mortality among patients with SMDs suggest indirectly that patients with SMDs are less likely to receive good quality somatic care. In addition to the unsatisfactory treatment of somatic care, the results highlight the challenges of organising health services. In light of our findings, a better integration of psychiatric and somatic services, as well as substance use and health services seems justified. Moreover, the excess mortality has increased over time, suggesting that patients with SMDs have not benefited from improvements in health care to the same extent as the general population. The results on increasing avoidable mortality among women in Finland suggest a deterioration in access to and/or the quality of somatic health services for patients with serious mental disorders over the last 20 years. Somatic diseases in mental health patients should be more carefully taken into account when designing mental and somatic health care services.

## Supporting Information

S1 Dataset Age-standardised mortality rates. Age-standardised mortality rates (/100 000 py) among the total population and patients with severe mental disorders (SMD) by gender and cause of death groups in 1996–2010 in Finland.
(XLSX)

S2 Dataset The rate ratios of the excess mortality. The rate ratios (RR) and 95% confidence intervals of the excess mortality in patients with severe mental disorders (SMD) compared to the total population by gender, cause-of-death groups, and the SMD categories (patients with psychotic disorders [PD], psychoactive substance use disorders [PSD], and mood disorders [MD]) in the periods 1996–98, 1999–2001, 2002–04, 2005–07 and 2008–10 in Finland.
(XLSX)

S3 Dataset The sensitivity analyses for the rate ratios of the excess mortality. The sensitivity analyses for the rate ratios (RR) and 95% confidence intervals of the excess mortality in patients with severe mental disorders (SMD) compared to the total population by gender, cause-of-death groups, and the SMD categories (patients with psychotic disorders [PD], psychoactive substance use disorders [PSD], and mood disorders [MD]) in the periods 1996–98, 1999–2001, 2002–04, 2005–07 and 2008–10 in Finland. Only patients with hospitalisations due to SMDs from a preceding period of five years or during the particular study year included.
(XLSX)

S4 Dataset The rate ratios of the excess mortality in patients with psychotic disorders (PD): patients comorbid with psychoactive substance use disorders (PSD) compared to patients without PSDs. The rate ratios (RR) and 95% confidence intervals of the excess mortality in patients with PDs comorbid with PSDs compared to patients with PDs without PSDs by gender and cause-of-death groups in the periods 1996–98, 1999–2001, 2002–04, 2005–07 and 2008–10 in Finland.
(XLSX)

## References

[1] De Hert M, Correll CU, Bobes J, Cetkovich-Bakmas M, Cohen D, Asai I, et al Physical illness in patients with severe mental disorders. I. Prevalence, impact of medications and disparities in health care. World Psychiatry. 2011;10(1): 52–77. 21379357 10.1002/j.2051-5545.2011.tb00014.x PMC3048500

[2] Penninx B, Milaneschi Y, Lamers F, Vogelzangs N. Understanding the somatic consequences of depression: biological mechanisms and the role of depression symptom profile. BMC Medicine. 2013;11(1): 129. 23672628 10.1186/1741-7015-11-129 PMC3661358

[3] Björkenstam E, Ljung R, Burström B, Mittendorfer-Rutz E, Hallqvist J, Weitoft GR. Quality of medical care and excess mortality in psychiatric patients—a nationwide register-based study in Sweden. BMJ Open. 2012;2(1). 10.1136/bmjopen-2011-000778 PMC3289986 22368297

[4] Ajetunmobi O, Taylor M, Stockton D, Wood R. Early death in those previously hospitalised for mental healthcare in Scotland: a nationwide cohort study, 1986–2010. BMJ Open. 2013;3(7). 10.1136/bmjopen-2013-002768 PMC3731727 23901025

[5] Regier DA, Farmer ME, Rae DS, Locke BZ, Keith SJ, Judd LL, et al Comorbidity of mental disorders with alcohol and other drug abuse: Results from the epidemiologic catchment area (eca) study. JAMA. 1990;264(19): 2511–2518. 2232018

[6] Havassy BE, Alvidrez J, Owen KK. Comparisons of Patients With Comorbid Psychiatric and Substance Use Disorders: Implications for Treatment and Service Delivery. Am J Psychiatry. 2004;161(1): 139–145. 14702262 10.1176/appi.ajp.161.1.139

[7] Maynard C, Cox GB, Hall J, Krupski A, Stark KD. Substance use and five-year survival in Washington State mental hospitals. Administration and policy in mental health. 2004;31(4): 339–345. 15285209 10.1023/b:apih.0000028896.44429.ca

[8] Brown S, Barraclough B, Inskip H. Causes of the excess mortality of schizophrenia. The British journal of psychiatry. 2000;177(3): 212–217. 11040880 10.1192/bjp.177.3.212

[9] Hamer M, Stamatakis E, Steptoe A. Psychiatric Hospital Admissions, Behavioral Risk Factors, and All-Cause Mortality: The Scottish Health Survey. Arch Intern Med. 2008;168(22): 2474–2479. 10.1001/archinte.168.22.2474 19064832

[10] Kisely S, Sadek J, MacKenzie A, Lawrence D, Campbell LA. Excess cancer mortality in psychiatric patients. Can J Psychiatry. 2008;53(11): 753–761. 19087469 10.1177/070674370805301107

[11] Saha S, Chant D, McGrath J. A systematic review of mortality in schizophrenia: Is the differential mortality gap worsening over time? Archives of General Psychiatry. 2007;64(10): 1123–1131. 17909124 10.1001/archpsyc.64.10.1123

[12] Tidemalm D, Waern M, Stefansson C, Elofsson S, Runeson B. Excess mortality in persons with severe mental disorder in Sweden: a cohort study of 12 103 individuals with and without contact with psychiatric services. Clinical Practice and Epidemiology in Mental Health. 2008;4(1): 23. 18854034 10.1186/1745-0179-4-23 PMC2576252

[13] Wahlbeck K, Westman J, Nordentoft M, Gissler M, Laursen TM. Outcomes of Nordic mental health systems: life expectancy of patients with mental disorders. The British Journal of Psychiatry. 2011;199(6): 453–458. 10.1192/bjp.bp.110.085100 21593516

[14] Kisely S, Campbell LA, Wang Y. Treatment of ischaemic heart disease and stroke in individuals with psychosis under universal healthcare. The British journal of psychiatry. 2009;195(6): 545–550. 10.1192/bjp.bp.109.067082 19949207

[15] Lawrence D, Kisely S. Inequalities in healthcare provision for people with severe mental illness. J Psychopharmacol. 2010;24(4): 61–68. 10.1177/1359786810382058 20923921 PMC2951586

[16] Nolte E, McKee CM. Does healthcare save lives? Avoidable mortality revised. 2004.

[17] Rutstein DD, Berenberg W, Chalmers TC, Child CG, Fishman AP, Perrin EB, et al Measuring the quality of medical care. New England Journal of Medicine. 1976;294(11): 582–588. 942758 10.1056/NEJM197603112941104

[18] Nolte E, McKee CM. Measuring the health of nations: Updating an earlier analysis. Health Affairs (Project Hope). 2008;27(1): 58–71. 18180480 10.1377/hlthaff.27.1.58

[19] Lawrence DM, Holman CDJ, Jablensky AV, Hobbs MST. Death rate from ischaemic heart disease in Western Australian psychiatric patients 1980–1998. The British journal of psychiatry. 2003;182(1): 31–36. 12509315 10.1192/bjp.182.1.31

[20] Räsänen S, Hakko H, Viilo K, Meyer-Rochow BV, Moring J. Avoidable mortality in long-stay psychiatric patients of Northern Finland. Nordic Journal of Psychiatry. 2005;59(2): 103–108. 16195106 10.1080/08039480510022909

[21] Hoang U, Goldacre MJ, Stewart R. Avoidable mortality in people with schizophrenia or bipolar disorder in England. Acta Psychiatr Scand. 2013;127(3): 195–201. 10.1111/acps.12045 23216065

[22] Manderbacka K, Arffman M, Sund R, Haukka J, Keskimäki I, Wahlbeck K. How does a history of psychiatric hospital care influence access to coronary care: a cohort study. BMJ Open. 2012;2(2). 10.1136/bmjopen-2012-000831 PMC3323812 22492387

[23] Page A, Tobias M, Glover J, Wright C, Hetzel D, Fisher E. Australian and New Zealand atlas of avoidable mortality Australia: Adelaide: PHIDU, University of Adelaide; 2006.

[24] Clayton D, Hills M. Statistical Models in Epidemiology New York: Oxford University Press; 1993.

[25] Corten P, Ribourdouille M, Hermann P, Rorsman B, Sims A. Epidemiological survey of the "natural" mortality in psychiatry. Acta Psychiatr Belg. 1988;88(5–6): 349–371. 3079167

[26] Leucht S, Burkard T, Henderson J, Maj M, Sartorius N. Physical illness and schizophrenia: a review of the literature. Acta Psychiatr Scand. 2007;116(5): 317–333. 17919153 10.1111/j.1600-0447.2007.01095.x

[27] Westman J, Hällgren J, Wahlbeck K, Erlinge D, Alfredsson L, Ösby U. Cardiovascular mortality in bipolar disorder: a population-based cohort study in Sweden. BMJ Open. 2013;3(4). 10.1136/bmjopen-2012-002373 PMC3641504 23604348

[28] Vuorenkoski L, Mladovsky P, Mossialos E. Finland: Health system review. Health Systems in Transition. 2008;10(4): 1–168. 31596240

[29] Ringbäck Weitoft G, Gullberg A, Rosen M. Avoidable mortality among psychiatric patients. Social psychiatry and psychiatric epidemiology. 1998;33(9): 430–437. 9766169 10.1007/s001270050076

[30] Pirkola S, Sohlman B, Heilä H, Wahlbeck K. Reductions in Postdischarge Suicide After Deinstitutionalization and Decentralization: A Nationwide Register Study in Finland. PS. 2007;58(2): 221–226. 10.1176/ps.2007.58.2.221 17287379

[31] Gissler M, Haukka J. Finnish health and social welfare registers in epidemiological research. Norsk Epidemiologi. 2004;14(1): 113–120.

[32] Sund R. Quality of the Finnish Hospital Discharge Register: A systematic review. Scandinavian Journal of Public Health. 2012;40(6): 505–515. 10.1177/1403494812456637 22899561

[33] Lahti RA, Penttilä A. The validity of death certificates: routine validation of death certification and its effects on mortality statistics. Forensic Sci Int. 2001;115(1–2): 15–32. 11056267 10.1016/s0379-0738(00)00300-5

